# Lyophilized Symbiotic Mitigates Mucositis Induced by 5-Fluorouracil

**DOI:** 10.3389/fphar.2021.755871

**Published:** 2021-12-10

**Authors:** Bruna Savassi, Bárbara F. Cordeiro, Sara H. Silva, Emiliano R. Oliveira, Giovanna Belo, Alessandra Gomes Figueiroa, Maria Izabel Alves Queiroz, Ana Maria Caetano Faria, Juliana Alves, Tales Fernando da Silva, Gabriela Munis Campos, Erick A. Esmerino, Ramon S. Rocha, Monica Q. Freitas, Marcia C. Silva, Adriano G. Cruz, Kátia Duarte Vital, Simone O.A. Fernandes, Valbert N. Cardoso, Leonardo Borges Acurcio, Gwénaël Jan, Yves Le Loir, Alfonso Gala-Garcia, Fillipe Luiz R. do Carmo, Vasco Azevedo

**Affiliations:** ^1^ Instituto de Ciências Biológicas, Universidade Federal de Minas Gerais (UFMG), Belo Horizonte, Brazil; ^2^ Faculdade de Veterinária, Universidade Federal Fluminense (UFF), Niterói, Brazil; ^3^ Departamento de Alimentos, Ciência e Tecnologia Do Rio de Janeiro (IFRJ), Instituto Federal de Educação, Rio de Janeiro, Brazil; ^4^ Departamento de Análises Clínicas e Toxicológicas, Universidade Federal de Minas Gerais (UFMG), Belo Horizonte, Brazil; ^5^ INRAE, STLO, Institut Agro, Agrocampus Ouest, Rennes, France; ^6^ Faculdade de Odontologia, Universidade Federal da Bahia (UFBA), Salvador, Brazil

**Keywords:** probiotic, chemotherapy, prebiotic, immunomodulant effects, symbiotic

## Abstract

Mucositis is an adverse effect of cancer chemotherapies using 5-Fluorouracil (5-FU). It is characterized by mucosal inflammation, pain, diarrhea, and weight loss. Some studies reported promising healing effects of probiotic strains, when associated with prebiotics, as adjuvant treatment of mucositis. We developed a lyophilized symbiotic product, containing skimmed milk, supplemented with whey protein isolate (WPI) and with fructooligosaccharides (FOS), and fermented by *Lactobacillus casei* BL23, *Lactiplantibacillus plantarum* B7, and *Lacticaseibacillus rhamnosus* B1. In a mice 5-FU mucositis model, this symbiotic lyophilized formulation was able to reduce weight loss and intestinal permeability. This last was determined *in vivo* by quantifying blood radioactivity after oral administration of 99mTc-DTPA. Finally, histological damages caused by 5-FU-induced mucositis were monitored. Consumption of the symbiotic formulation caused a reduced score of inflammation in the duodenum, ileum, and colon. In addition, it decreased levels of pro-inflammatory cytokines IL-1β, IL-6, IL-17, and TNF-α in the mice ileum. The symbiotic product developed in this work thus represents a promising adjuvant treatment of mucositis.

## Introduction

Mucositis consists of an inflammation, mainly of the small bowel, that affects individuals submitted to cancer chemotherapy treatments, such as 5-Flourouracil (5-FU) (Sonis, 2004). It includes mucosal injury, inflammation, diarrhea, and weight loss. It may lead to mucosal lesions and/or ulcerations throughout the gastrointestinal tract (Rodríguez-Caballero et al., 2012). Mucositis markers include the presence of leukocyte infiltrate in the lamina propria, degenerate enterocytes (Ciorba et al., 2016), accumulation of neutrophils and eosinophils (Antunes et al., 2016), increased degeneration of goblet cells (Stringer, 2013), as well as atrophy of villi (Chang et al., 2012).

There is presently no effective treatment for the prevention or alleviation of symptoms of mucositis. Furthermore, the use of chemotherapeutics causes severe dysbiosis (imbalance in the intestinal microbiota) which in turn worsens intestinal inflammation (van der Velden et al., 2014). In this context, development of alternative or adjuvant treatments is needed. Indeed, the use of probiotics as promising candidates for adjuvant treatment of mucositis recently attracted attention (Carvalho RD. et al., 2017). Selected lactic acid bacteria (LAB) strains were reported as probiotics with beneficial effects mediated by different mechanisms of action and offer new perspectives for the development of adapted functional foods (Carvalho RDO. et al., 2017; Eales et al., 2017; Tang et al., 2017). Thus, studies have been carried out to evaluate the potential of such probiotic strains, associated with prebiotics, as possible symbiotic treatments of mucositis (Bastos et al., 2016).

Administration of lactobacilli strains, or of probiotic formulations, can, in pre-clinical models, alleviate experimental mucositis and prevent weight loss, diarrhea, and intestinal damages (Justino et al., 2015; Cordeiro et al., 2018; Do Carmo et al., 2019). As an example, Cordeiro and collaborators showed that the *L. casei* BL23 strain, when grown in milk supplemented with whey protein isolate, was able to mitigate inflammation in 5-FU-induced mucositis in mice (Cordeiro et al., 2018). Moreover, Galdino et al. (2018) demonstrated that Fructooligosaccharides (FOS), recognized as prebiotic, were able to reduce mucosal damages in such a model (Galdino et al., 2018)**.** Trindade et al. (2018) further reported that the use of the symbiotic Simbioflora^®^ reduced intestinal injury in such a model (Trindade et al., 2018)**.** Products combining probiotics and prebiotics are called symbiotic. They may contain one or more probiotic strain(s) and one or more prebiotic compound(s) (Flesch et al., 2014)**.** They are designed to favor synergy between the combined elements, providing the consumer with the beneficial effects of this association (Flesch et al., 2014). Several studies carried out with symbiotics highlighted effects such as reduction of pro-inflammatory cytokines (Ishikawa et al., 2005), stimulation of the immune system (Raizel et al., 2011), and reduction of intestinal infections and intestinal inflammation (Santos et al., 2015)**.** Futher efforts focused on the quest for new strains, or consortia thereof, to be used as adjuvants in the treatment of mucositis (Picó-Monllor and Mingot-Ascencao, 2019; Shu et al., 2020). In this quest, new candidate strains *Lactiplantibacillus plantarum* B7 and *Lacticaseibacillus rhamnosus* D1 may open new perspectives. They were recently shown to prevent infections by *Salmonella enterica* serovar Typhimurium in BALB/c mice and in germ-free-mice, including clinical manifestations such as tissue damages at the level of ileum (Acurcio L. B. et al., 2017, Acurcio et al., 2017 LB.; Valente et al., 2019). This protective effect being anti-inflammatory, these strains seem good candidates to be investigated in the context of mucositis.

The aim of this work was thus to develop a symbiotic and lyophilized product, based on milk, supplemented with WPI and FOS, fermented by strains *L. casei* BL23, *L. plantarum* B7, and *L. rhamnosus* B1, which would be able to reduce the intestinal inflammation, to control the pro-inflammatory immune response, and to decrease intestinal permeability, in a murine model of mucositis induced by 5-FU.

## Materials and Methods

### Bacterial Strains and Culture Conditions

The bacterial strain *L. casei* BL23 is part of the UMR1219 MICALIS INRA-AgroParisTech collection, Jouy-en-Josas, France. The strains *L. plantarum* B7 and *L. rhamnosus* D1 were provided by Professor Leonardo Acúrcio of Microorganisms and Ecology Laboratory of Physiology, Universidade Federal de Minas Gerais. An aliquot of the bacterial strains *L. casei* BL23, *L. plantarum* B7, and *L. rhamnosus* D1 were first inoculated (2% v/v) in MRS culture medium (deMan, Rogosa, and Sharpe) for 24 h at 37°C. Aliquots of bacterial cultures from each strain were then inoculated into 12% w/v low-fat milk medium (0.1% w/v yeast extract, 2% w/v glucose) supplemented or in the absence of whey protein isolate (WPI) 30% w/v. After growth (24 h 37°C), a 1 ml aliquot of each inoculum was removed to assess colony forming unit (CFU) count. Subsequently, 500 ml of each sample along with 500 ml of skimmed milk supplemented with WPI without the presence of bacteria (Matrix), were refrigerated and lyophilized in LH modelo 0601 (LIOMEAL—LBR Liofilização do Brasil). Posteriorly, for 5-FU-induced mucositis mice model, all three strains fermented beverages (*L. casei* BL23, *L. plantarum* B7, and *L. rhamnosus* D1) subjected to lyophilization were homogenized in a 1:1:1(g) ratio and, subsequently, added with FOS (NewNutrition^®^) in a sterile environment also at a ratio of 1:1(g). The product composed of three strains lyophilized in a matrix and supplemented with FOS was called Symbiotic.

### Physicochemical Analyses and Bioactivity

The determination of moisture, protein, and fat content were evaluated according to what was previously described (BRASIL, 2006). To determine the moisture content, we oven-dry 5 g of a sample at 100–105°C, for 24 h. For protein content quantification and fat levels, we realized the Kjeldahl and Gerber method, respectively (Cordeiro et al., 2021). All results were expressed as g/100 g. The bioactive peptides levels were determinated evaluate the angiotensin I-converting enzyme inhibition (ACEI), antioxidant activity (DPPH), and α-amylase and α-glucosidase inhibition. For angiotensin I-converting enzyme inhibitory (ACEI) calculate we used the spectrophotometric assay, according to Konrad et al. (2014). For 2,2-diphenyl-1-picrylhydrazyl (DPPH) measurmeant, we used the radical-scavenging method previously described (Lee et al., 2016). Finally, for measurement of α-glucosidase and α-amylase inhibitory activities of lyophilized formulations we used the protocol describe by Grom et al. (2020).

### Evaluation of Probiotics Properties of the Formulations in Mucosite Mice Models

#### Animals

Conventional BALB/c mice (female) between 6 and 8 weeks of age, obtained at Universidade Federal de Minas Gerais (UFMG–Belo Horizonte, Brazil), were used. All mice were kept in a room with temperature-controlled and standard chow diet and *ad libitum* access to water. This study was approved by the Ethics Committee on Animal Experimentation of the Federal University of Minas Gerais (379/2018).

#### Experimental Set-Up

For probiotic treatment, mice were gavaged daily with 500 mg (per day per animal) of dried product resuspended in PBS pH 7.4 until dissolution (500 µl for maximum final volume) for 13 days. The maximal volume given daily by gavage was set according to the good practice guide to the administration of substances (Diehl et al., 2001). To induce the mucositis disease, on day 11, mice of inflamed groups received a single injection of 5-FU (Fauldfluor—Libbs) (300 mg/kg, intraperitoneally). An injection of saline (NaCl 0.9%) was used in control groups. After 72 h of this mucositis induction, all mice were euthanized (Carvalho RD. et al., 2017). A longitudinal abdominal incision was performed to remove the intestine for further analyses. The mice were weighed daily. BALB/c mice were divided into five groups. The non-inflamed control (naive) and inflamed control (5-FU) groups received 500 µl of PBS pH 7.4, the other three groups received the dose of 5-FU, and were gavaged with 500 mg of samples resuspended in PBS pH 7.4, i.e., the matrix control group was gavaged with the lyophilization matrix (with FOS). The *L. casei* BL23 group and the Symbiotic group were gavaged with probiotic formulations containing in addtion to WPI (lyophilization matrix) and FOS (250 mg per dose), either BL23 (10^9^ CFU) or the 3 strains (10^9^ CFU). The maximal volume given daily by gavage was set according to the good practice guide to the administration of substances (Diehl et al., 2001). Each group contained 18 animals.

#### Histopathological Analysis

The distal portion of the duodenum, jejunum, and ileum from mice was collected and prepared for histomorphological analysis. For that, tissues were immersed in 4% buffered formaldehyde solution and then the material was embedded in paraffin, and a 4-μm section of each sample was placed on a glass slide and stained with hematoxylin-eosin (HE). The histological score was done by a pathologist, using Soares et al. (2008) protocol. In this protocol, the intensity of the infiltrate of mononuclear and polymorphonuclear cells in the lamina propria of the duodenum, jejunum, and ileum, the presence of ulceration and erosion and changes in mucosal architecture were measured (Soares et al., 2008). For each parameter a classification was given according to the severity of the lesion in the tissues: absent (0), mild (1), moderate (2), and severe (3). For morphometric analysis, 10 images of the ileum of each animal were randomly captured and analyzed using ImageJ software (version 1.8.0). Additional cuts in the paraffinized samples from duodenum, jejunum, and ileum were stained by the Periodic Acid-Schiff (PAS) to determine the number of goblet cells in the tissues (Prisciandaro et al., 2011). Ten random field images of each sample were made using the 40x objective and the intact goblet cells were counted using ImageJ software (version 1.8.0) and expressed as the number of cells per high-power field (hpf) (40x, 108.2 μm^2^) (Cordeiro et al., 2018).

#### Intestinal Permeability

To assess intestinal permeability, after 72 h of mucositis induction, a group of animals received 0.1 ml of diethylenetriaminepentaacetate acid (DTPA), labelled with 18.5 MBq of ^99m^technetium, by gavage. Four hours later, the blood was collected, placed in appropriate tubes for radioactive determination and weighing (de Barros et al., 2018). Results were calculated as percentage of dose per g of blood, by the following equation: % dose/g blood = (cpm in g of blood/cpm dose of standard) × 100 cpm (counts of radioactivity per minute) (Galdino et al., 2018; Do Carmo et al., 2019).

#### Gene Expression Analysis in the Mice Ileum

Fragments of 1 cm of ileum were collected and total RNA of samples was extracted using PureLink RNA Mini Kit (Thermo Fisher Scientific). The extraction protocol was done according to the manufacturer. To digest and remove residual genomic DNA of samples we used DNase I (Invitrogen; Waltham, MA) and Turbo DNA-free Kit (Ambion; Austin, TX). RNA quality was assessed using agarose gel and NanoDrop^®^ ND-1000 (260/230 ratio). To prepare the cDNA libraries we used the High-Capacity cDNA Reverse Transcription kit (Applied Biosystems; Foster City, CA). Quantitative PCR (qPCR) was determined using iTaq universal SYBR green supermix (Biorad; Hercules, CA) and gene specific-primers, were selected according to Do Carmo et al. (2019), for zonula occludes 1 and 2 (*zo-1* and *zo-2*, respectively), occludin (*ocln*), claudin-1 (*cln-1*), and claudin-5 (*cln-5*). For housekeeping genes, we encoded β-actin (*actβ*) and GAPDH (*gapdh*). The amplification cycles were performed as described: 95°C for 30 s, and 40 cycles of 95°C for 15 s and 60°C for 30 s on ABI PRISM 7900HT Sequence Detection System (Applied Biosystems). Results were expressed as a fold-change of expression levels, using the mean and standard deviations of target expression (2^−ΔΔCt^).

#### Ileum Tissue Preparation for Cytokine Quantification by ELISA

Pro- and anti-inflammatory cytokines were quantified by ELISA assay. Briefly, the ileum section of were weighed and homogenized (100 mg tissue/ml buffer) in PBS containing 0.05% Tween-20 (Sigma-Aldrich, St. Louis, MO), phenylmethylsulfonyl fluoride 0.1 mM (Sigma- Aldrich, St. Louis, MO), benzethonium chloride 0.1 mM (Sigma-Aldrich, St. Louis, MO), EDTA 10 mM (Synth, São Paulo, São Paulo, Brazil), and aprotinin A 20 KIU (Sigma-Aldrich, St. Louis, MO). Suspensions were centrifuged at 3.000 g for 10 min 12 and the supernatants were collected for dosage of IL-1β, IL-6, IL- 10, IL-17, TNF-α, and INF-γ cytokine according to the R and D Systems, Inc. protocols. The absorbance was measured at 492 nm using a Microplate Reader Model 680 (BIO-RAD). Samples from six animals per group were collected for the ELISA assay, homogenized, and three technical replicates performed.

#### Statistical Analyses

Data were performed using one-way ANOVA or two-way ANOVA followed by the Tukey or Sidak post-test. Graphs and statistical analyzes were analized using GraphPad Prism version 9.2.0 (332) for Windows (GraphPad Software, San Diego, CA). All results were presented as the mean ± standard deviation, and *p* < 0.05 was considered as statistically significant.

## Results

### Viability of *L. casei* BL23, *L. plantarum* B7, and *L. rhamnosus* D1 Strains Submitted to Lyophilization

The viability of strains *L. casei* BL23, *L. plantarum* B7, and *L. rhamnosus* D1, submitted to lyophilization, was measured by CFU couting after rehydration of the product. All three strains, cultivated in the formulations with or without WPI, reached populations greater than 2 × 10^9^ CFU/g. The population of *L. casei* BL23 was 4 × 10^9^ CFU/g and 3 × 10^9^ CFU/g (before and after lyophilization, respectively). That of *L. plantarum* B7 was 3.3 × 10^9^ CFU/g and 2.3 × 10^9^ CFU/g (before and after lyophilization, respectively). Similar results were observed with *L. rhamnosus* D1: 4.9 × 10^9^ CFU/g and 2.5 × 10^9^ CFU/g (before and after lyophilization, respectively).

### Proximate Composition and Bioactivity Compounds

The Matrix formulation, containing WPI and FOS, was used as a control in the analysis of proximate composition and bioactivity compounds. The proximal composition of the probiotic formulations is described in Table 1. Analyzed parameters were moisture, proteins, lipids, lactose, and ash. It was not possible to find significant differences between the fermented samples (*L. casei* BL23 or Symbiotic in the presence or absence of FOS). However, there was a significant difference between the Matrix control and the other formulations.

**TABLE 1:** Proximal composition of fermented milks.

Samples	Moisture	Protein	Fat	Lactose	Ash
**Matrix**	70.3 ± 0.85^b^	2.26 ± 0.02^b^	2.41 ± 0.19^b^	24.99 ± 0.83^b^	0.6 ± 0.0&^b^
***L. casei* BL23**	88.2 ± 0.05^a^	3.52 ± 0.24^a^	1.81 ± 0.19^a^	5.82 ± 0.16^a^	0.65 ± 0.06^a^
***L. casei* BL23 + FOS**	88.3 ± 0.04^a^	3.53 ± 0.91^a^	1.89 ± 0.28^a^	5.57 ± 0.18^a^	0.71 ± 0.07^a^
**Symbiotic**	89.7 ± 0.09^a^	3.54 ± 0.43^a^	1.88 ± 0.62^a^	4.22 ± 0.92^a^	0.66 ± 0.02^a^
**Symbiotic + FOS**	88.4 ± 0.02^a^	3.52 ± 0.21^a^	1.82 ± 0.51^a^	5.58 ± 0.70^a^	0.68 ± 0.06^a^

* Data are expressed as the mean ± standard deviation of at least 3 replicates.

^a–f^ Different letters in the same column indicate significant differences between samples (*p* < 0.05).

Table 2 shows the bioactive compounds in the formulations proposed in this study. Regarding the analyzed bioactives: antioxidant potential (DPPH), inhibition of the enzyme converting angiotensin ACE, inhibition of α-amylase, and inhibition of α—glucosidase, the Symbiotic lyophilized product showed the highest values with significant differences (*p* < 0.05), when compared to other lyophilized product. Moreover, there was a significant difference (*p* < 0.05) in the bioactives levels of the Symbiotic with FOS, with higher values, when compared to the Symbiotic formulation in the absence of FOS.

**TABLE 2:** Bioactive compounds of fermented milks.

Samples	DPPH Antioxydant	ACE Inhibition	α - amylase Inhibition	α - glucosidase Inhibition
**Matrix**	23.7 ± 0.32^d^	23.2 ± 1.76^e^	18.2 ± 0.31^e^	20.5 ± 0.38^e^
***L. casei* BL23**	23.1 ± 0.13^d^	37.8 ± 0.24^d^	26.7 ± 0.79^d^	32.1 ± 0.16^d^
***L. casei* BL23 + FOS**	34.1 ± 0.34^c^	43.1 ± 0.98^c^	39.2 ± 0.21^c^	49.8 ± 0.18^c^
**Symbiotic**	44.3 ± 0.09^b^	58.2 ± 1.10^b^	49.2 ± 0.45^b^	55.6 ± 0.92^b^
**Symbiotic + FOS**	55.6 ± 0.28^a^	62.1 ± 0.30^a^	62.1 ± 0.22^a^	68.9 ± 0.70^a^

* Data are expressed as the mean ± standard deviation of at least 3 replicates.

^a–f^ Different letters in the same column indicate significant differences between samples (*p* < 0.05). The DDPH, ACE, α - amylase and α—glucosidase was expressed in %.

### Symbiotic Reduces the Weight Loss in Mice with Mucositis

**FIGURE 1:**
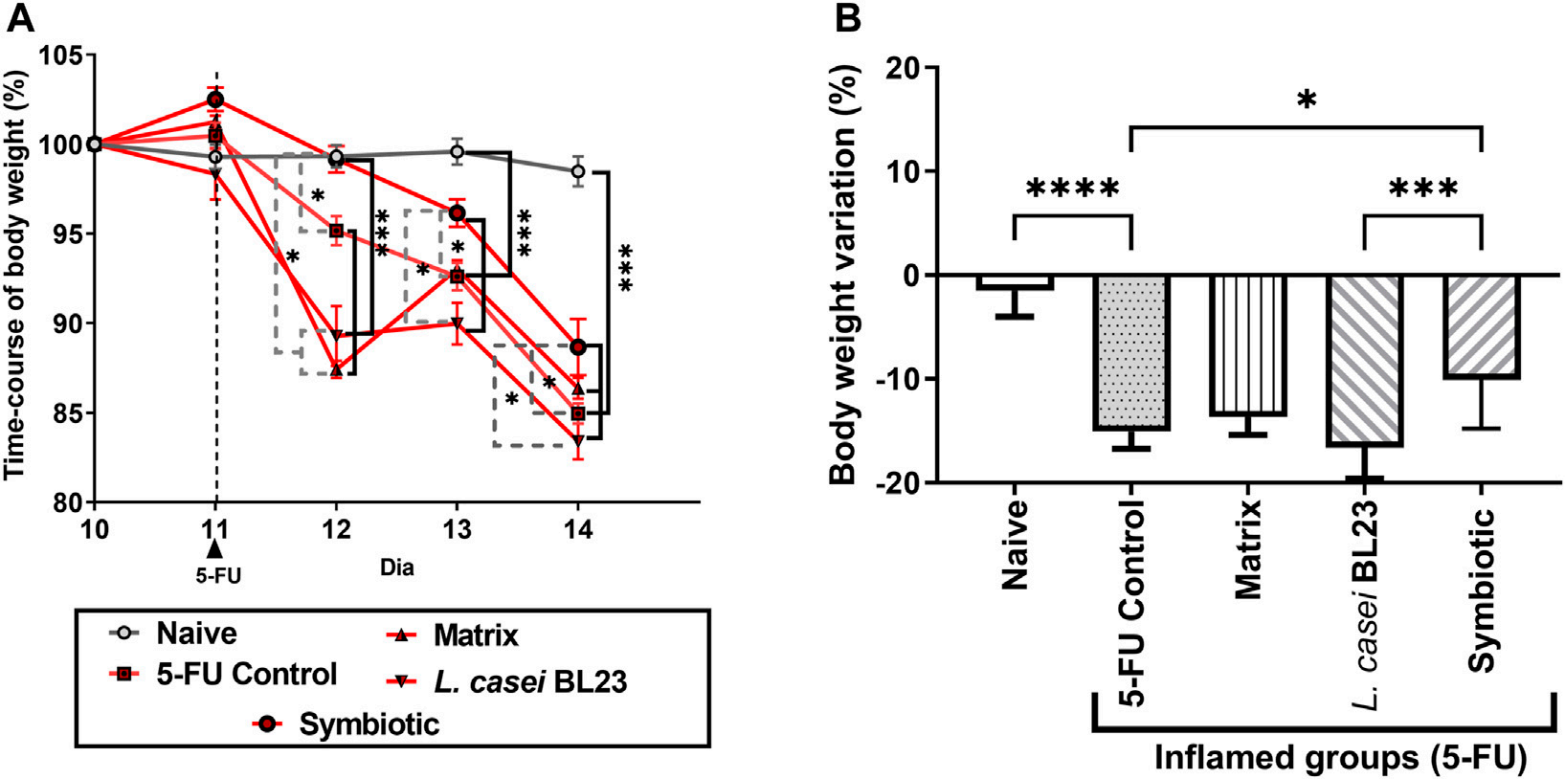
Probiotic lyophilized formulations can interfere with weight loss in mice with 5-FU-induced mucositis. Variation in body weight of mice (in percentage) over the last 5 experimental days **(A)** of animals that were gavaged with: PBS pH 7.4 (Naive group and 5-FU inflamed control group); 12% skimmed milk supplemented with 30% WPI and Fructooligosaccharide (Matrix); a formulation containing *L. casei* BL23, a formulation containing the mix of probiotics (**Symbiotic**). **(B)** Body weight variation (in percentage) observed after the last experimental day (14th day) considering the 10th experimental day as initial weight (100%). Animals gavaged with PBS was used as experimental controls. The one-way or two-way ANOVA test, followed by Sidak or Tukey post-test was used for the multiple comparisons between groups (n = 6–9). Asterisks represent statistically significant differences as follows: *p < 0.05; **p < 0.01; ***p < 0.001; ****p < 0.0001.

Figure 1 shows the time-course of mice body weight monitoring during the last five experimental days (10th to 14th day). During the pre-treatment period, prior to mucositis induction (1st to 10th day), there was no significant difference (data not shown) between the control groups (Naive and 5-FU) and the experimental groups (Matrix, *L. casei* BL23, Symbiotic). After the induction of mucositis, Figure 1A shows that the animals receiving the dose of 300 mg/kg of the chemotherapy 5-FU began to lose weight on the 12th experimental day, while bodyweight of control naïve mice remained constant. Moreover, all groups receiving 5-FU showed significant weight loss, when compared to the Naive group. The group of animals that received the Symbiotic treatment showed a significant difference (*p* < 0.05) in the daily variation of weight loss on the 12th and 13th experimental days. The peak of weight loss occurred on the 13th and 14th day of the experiment (Figure 1A). 5-FU administration, as shown in Figure 1B, induced weight loss in all experimental groups. However, the Symbiotic treatment significantly reduced (*p* < 0.05) the loss, compared to 5-FU (inflamed control group) and to *L. casei* BL23. We also observed a small weight loss in the Naive group without inflammation.

### Symbiotic Improves Mucosal Preservation in Small Intestine of the Inflamed Mice

**FIGURE 2:**
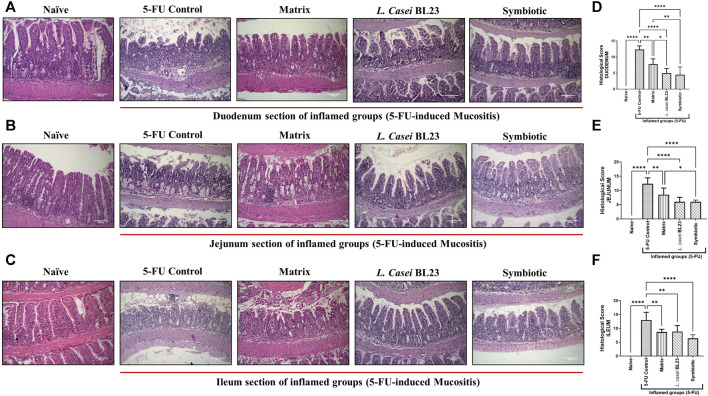
Probiotic lyophilized formulations can reduce inflammation in the duodenal, jejunum, and ileum section of 5-FU-induced mucositis mice. **(A, B, C)** Photomicrographs of the duodenal, jejunum, and ileum of BALB/c mice, stained in HE, induced or not to mucositis by 5-FU and those that received the probiotic lyophilized formulations. The animals were gavage with: PBS pH 7.4 (Naive group and 5-FU inflamed control group); 12% skimmed milk supplemented with 30% WPI and Fructooligosaccharide (Matrix); a formulation containing *L. casei* BL23, a formulation containing the mix of probiotics (Symbiotic). The photos show the ×20 magnification. Scale bar = 100 µm **(D, E, F)** Histopathological score of the duodenal, jejunum, and ileum section. Results were expressed as means ± standard deviation (n = 6–9). One-way ANOVA and Tukey post-hoc tests were used for multiple comparison. Asterisks represent statistically significant differences as follows: *p < 0.05; **p < 0.01; ***p < 0.001; ****p < 0.0001.

After euthanasia, the duodenal, jejunal, and ileal sections of the animals were collected and stained with HE and submitted to histological analysis to evaluate mucositis severity. Figures 2A–C show representative photos of the duodenum, jejunum, and ileum section, respectively. In the Naive group, no morphological change in duodenum, jejunum, and ileum sections was observed according to the parameters analyzed. Sections were devoid of inflammatory infiltrate and the general architecture of the mucosa remained unchanged. In the 5-FU inflamed control group, an increase in the infiltration of inflammatory cells in the lamina propria, submucosa, and muscle layer was observed in duodenum, jejunum, and ileum section. So was an increase in the thickness of the muscle layer, as well as a drastic change in the villi, being partially or completely destroyed. Furthermore, in some animals in the 5-FU inflamed group, the presence of ulceration and erosion was observed (not shown in the images). The group that received the Matrix showed moderate preservation of the architecture and height of the villi, with a partial destruction of the crypts and the presence of a moderate (mixed) inflammatory infiltrate, reaching the mucosa and submucosa. The group that received the *L. casei* BL23 formulation showed moderate mononuclear inflammatory infiltration, moderate to intense destruction of the crypts, and a moderate preservation of the villi architecture and height. In the group that received the Symbiotic formulation, discrete destruction and reduction of villi, moderate inflammatory infiltrate with mucosal and submucosal involvement, with moderate loss of crypts, can be observed. The histopathological score of the duodenum, jejunum, and ileum sections (Figures 2D–F, respectively) shows a reduction in the analyzed parameters with a significant difference between the 5-FU group and the groups treated with the Matrix, *L. casei* BL23, or Symbiotic (*p* < 0.01; *p* < 0.001; *p* < 0.001, respectively). In addition, the comparative analysis identified significant differences between the group treated with the Matrix, and the group treated with Symbiotic, for scores in duodenum and jejunum section (*p* < 0.05; *p* < 0.01, respectively), with a significant reduction of the histopathological score in the latter.

**FIGURE 3:**
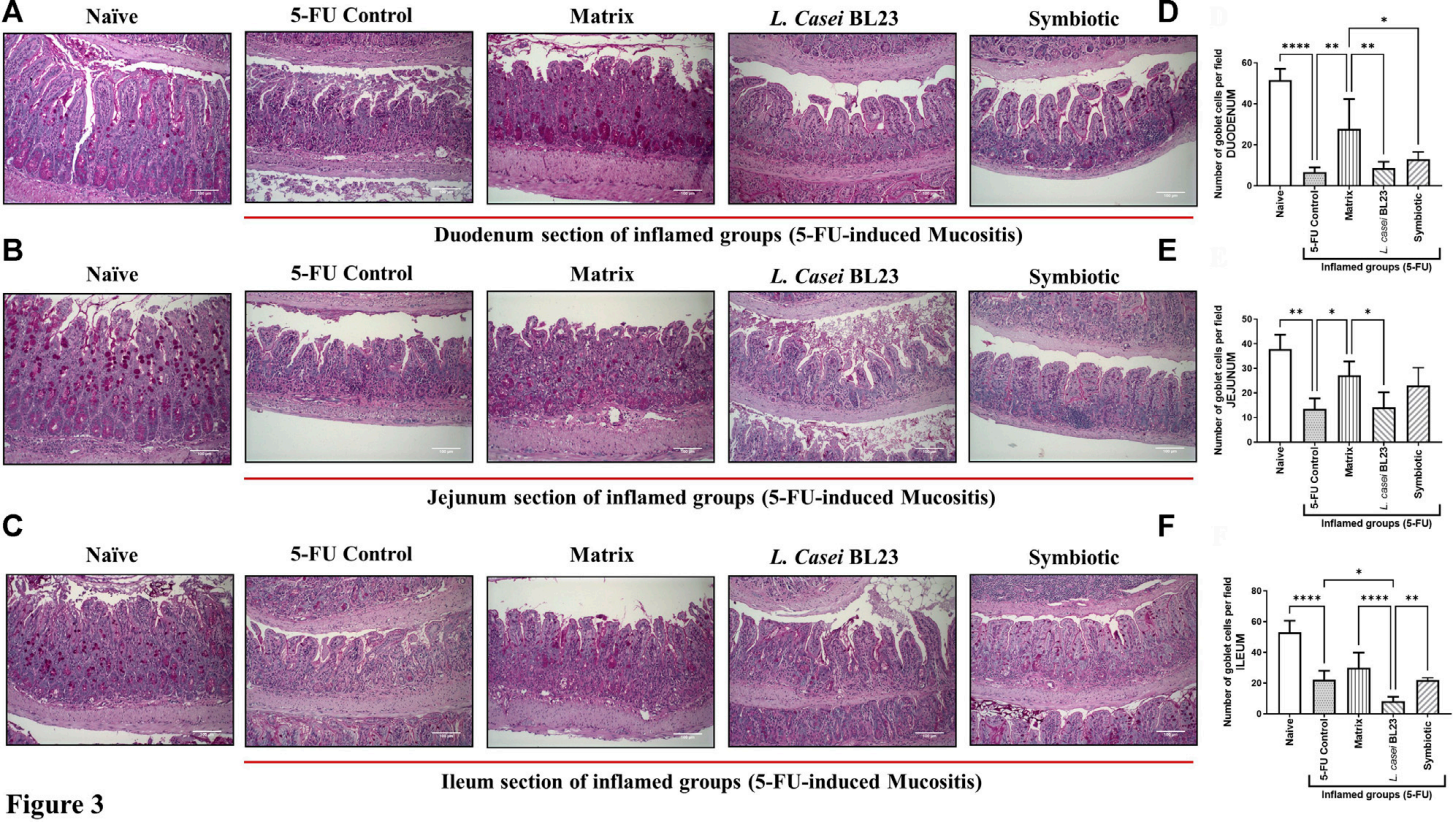
Probiotic lyophilized formulations can interfere in the population of goblet cells in the duodenal, jejunum, and ileum section of 5-FU-induced mucositis mice. **(A, B, C)** Photomicrographs of the duodenal, jejunum, and ileum, stained by PAS, of BALB/c mice submitted to 5-FU-induced mucositis and those that received the probiotic lyophilized formulations. The animals were gavage with: PBS pH 7.4 (Naive group and 5-FU inflamed control group); 12% skimmed milk supplemented with 30% WPI and Fructooligosaccharide (Matrix); a formulation containing *L. casei* BL23, a formulation containing the mix of probiotics (Symbiotic). The photos show the ×20 magnification. Scale bar = 100 µm **(D, E, F)** Quantification of goblet cells in the section of the duodenal, jejunum, and ileum by field of highest magnification (×40; 108.2 µm^2^). Results were expressed as means ± standard deviation (n = 6–9). One-way ANOVA and Tukey post-hoc tests were used for multiple comparison. Asterisks represent statistically significant differences as follows: *p < 0.05; **p < 0.01; ***p < 0.001; ****p < 0.0001.

As expected, the 5-FU induced mucositis in this mice model triggers substantial decrease in goblet cells number 6.66, 13.47, 22.30 goblet cell/hpf in duodenum, jejunum, and ileum section, respectively (Figures 3A–C for) when compared to the groups injected with 0.9% saline, 51.4, 37.9, 52.93 goblet cell/hpf in duodenum, jejunum, and ileum section, respectively. In the other hand, *L. casei* BL23 and Symbiotic treatment did not prevent the degeneration of goblet cells in the mice duodenum, jejunum, and ileum section. However, only the Matrix was able to significantly reduce the degeneration of goblet cells in the duodenum and jejunum (27.8, 27.2 goblet cell/hpf, *p* < 0.05; *p* < 0.01, respectively), not in the ileum.

### Symbiotic Prevents Increase in Gut Permeability

**FIGURE 4:**
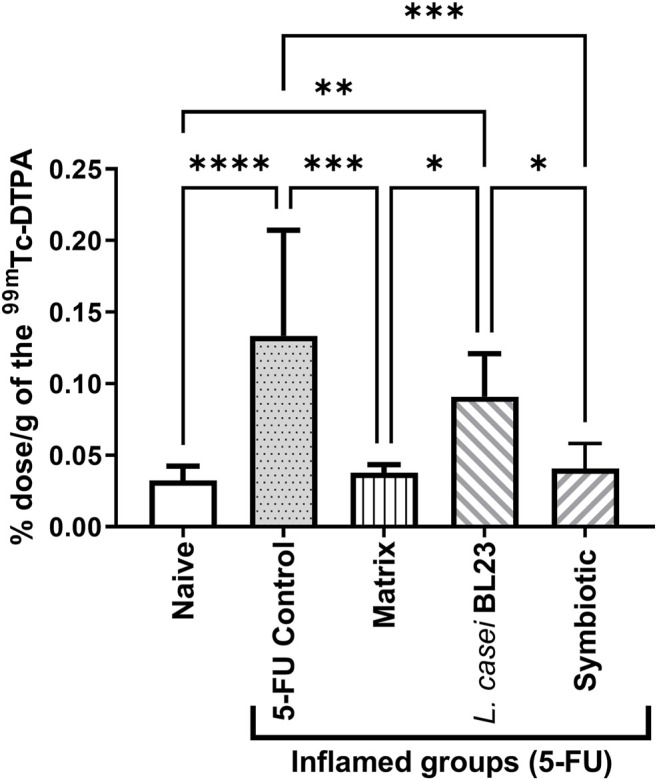
Lyophilized probiotic formulations can decrease intestinal permeability in mice with 5-FU-induced mucositis. Intestinal permeability was measured 72 h after mucositis induction by determining Technetium-99 m radioactivity (99mTc-DTPA) in mouse blood. The animals were gavage with: PBS pH 7.4 (Naive group and 5-FU inflamed control group); 12% skimmed milk supplemented with 30% WPI and Fructooligosaccharide (Matrix); a formulation containing L. casei BL23, a formulation containing the mix of probiotics (Symbiotic). Means and standard deviations were calculated from an independent experiment for each of the 9 animals per group. Asterisks represent statistically significant differences between the strains and were indicated as follows: *p < 0.05; **p < 0.01; ***p < 0.001 and ****p < 0.0001.

Intestinal permeability was evaluated after oral gavage of mice with radiolabelled diethylenetriaminepentaacetate (^99m^Tc-DTPA), followed by quantification of radioactivity in the animal´s blood. As expected, 5-FU injection significantly increased intestinal permeability (*p* < 0.001), compared to the Naive control group (Figure 4). There was no significant difference between the group treated with the *L. casei* BL23 and the 5-FU control group. However, animals treated with Matrix and Symbiotic exhibited significantly decreased (*p* < 0.001) intestinal permeability, compared to the 5-FU inflamed control group. Furthermore, there was no difference between the Naive, Matrix, and Symbiotic groups. Additionally, when the Matrix and Symbiotic groups were compared to the *L. casei* BL23 group, a significant reduction in intestinal permeability values was observed (*p* < 0.05).

### Symbiotic Increases Expression of Epithelial Barriers Genes

**FIGURE 5:**
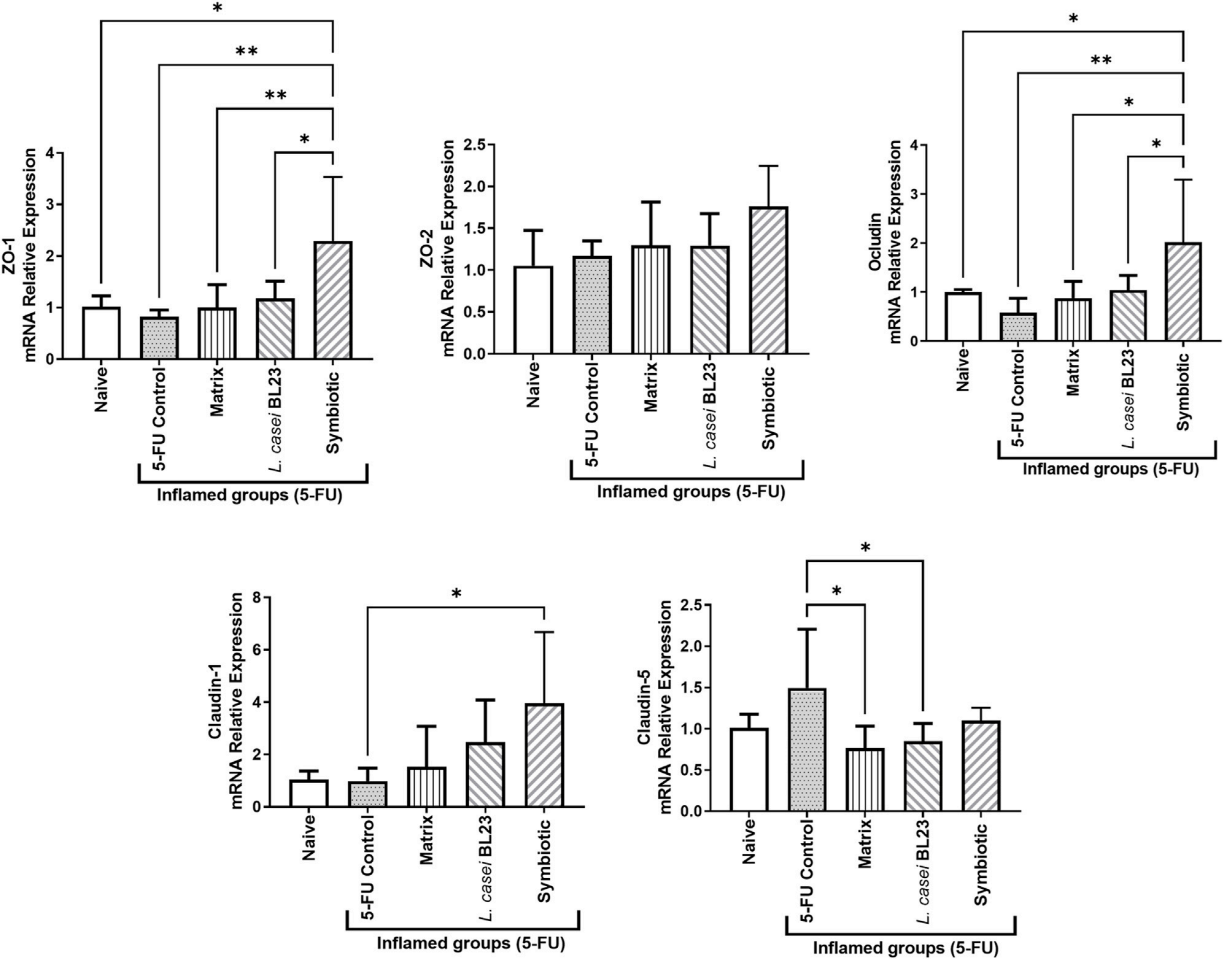
Probiotic lyophilized formulations can modulate gene expression of epithelial barrier genes in the ileum section of mice with 5-FU-induced mucositis. Gene for zonula occludes 1 and 2 (zo-1 and zo-2, respectively), occludin (ocln), and claudin-1 (cln-1) were measured by RT-qPCR. The animals were gavage with: PBS pH 7.4 (Naive group and 5-FU inflamed control group); 12% skimmed milk supplemented with 30% WPI and Fructooligosaccharide (Matrix); a formulation containing *L. casei* BL23, a formulation containing the mix of probiotics (Symbiotic). Means and standard deviations are calculated from 3 animals per group, from 2 independent repetitions and each quantification was performed in duplicate (technical duplicate). Asterisks represent statistically significant differences between strains and were indicated as follows: *p < 0.05; **p < 0.01; ***p < 0.001 and ****p < 0.0001.

Among Naïve, 5-FU (inflamed control group), Matrix, and *L. casei* BL23 groups, no difference was found between groups in the expression of genes *zo-1*, *zo-2*, *claudin-1,* and *occluding* (Figure 5). However, the Symbiotic treatment was able to significantly increase the expression of the genes zo-1 (*p* < 0.01), occludin (*p* < 0.05), and claudin-1 (*p* < 0.05), when compared to the 5-FU inflamed control groups. It is noteworthy that expression of the ZO-1 and Occludin genes in the Symbiotic group was significantly higher, when compared to Matrix and *L. casei* BL23 groups, respectively, *zo-1* (*p* < 0.01; *p* < 0.05), *ocludin* (*p* < 0.05; *p* < 0.05).

### Symbiotic Modulates Anti-Inflammatory Cytokines in Mice Ileum

**FIGURE 6:**
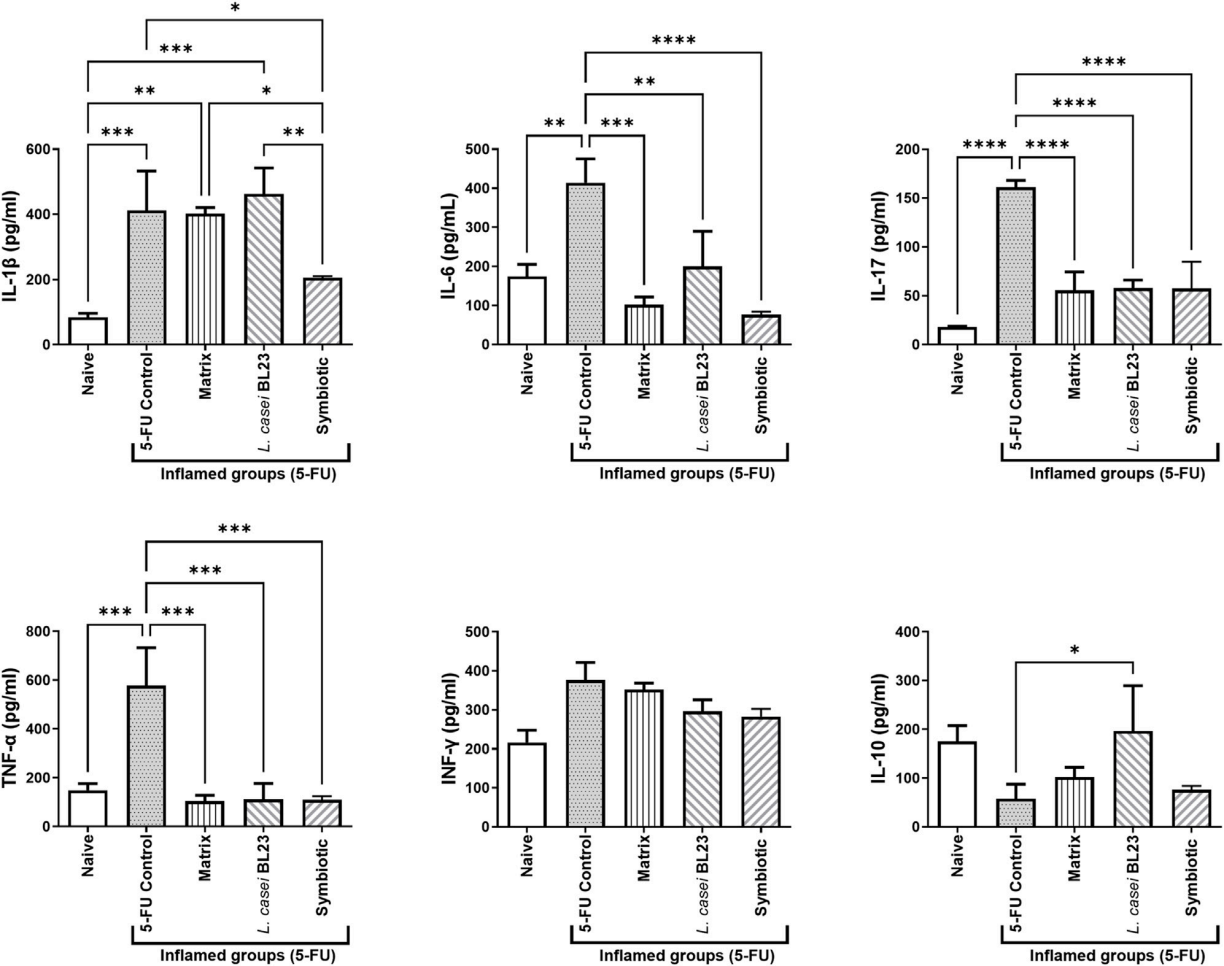
Probiotic lyophilized formulations can modulate cytokine production in the ileum section of mice with 5-FU-induced mucositis. Cytokine levels (A IL-1β, IL-6, IL- 10, IL-17, TNF-α, and INF-γ ratio were determined in mouse ileum tissue supernatant by ELISA. The animals were gavaged with: PBS pH 7.4 (Naive group and 5-FU inflamed control group); 12% skimmed milk supplemented with 30% WPI and Fructooligosaccharide (Matrix); a formulation containing L. casei BL23, a formulation containing the mix of probiotics (Symbiotic). Means and standard deviations are calculated from 3 animals per group, from 3 independent repetitions, and each quantification was performed in triplicate (technical triplicates). Asterisks represent statistically significant differences between strains and were indicated as follows:*p < 0.05; **p < 0.01; ***p < 0.001 and ****p < 0.0001.

As shown in Figure 6, the cytokines IL-1β, IL-6, IL-17, and TNF-α were significantly enhanced (*p* < 0.001; *p* < 0.05; *p* < 0.0001; *p* < 0.001, respectively) in the ileum of animals administered with 5-FU (inflamed control group), when compared to the Naïve control group. However, Symbiotic treatment was able to reduce significantly cytokines levels of IL-1β, IL-6, IL-17, TNF-α (*p* < 0.05; *p* < 0.0001; *p* < 0.0001; *p* < 0.001, respectively) compared to the 5-FU control group. In addition, the Symbiotic group reduced significantly (*p* < 0.05) the levels of IL-1β, when compared to the Matrix group. The Matrix and *L. casei* BL23 treatments were also able to reduce significantly cytokines levels of IL-6, IL-17, and TNF-α, compared to the 5-FU group. No difference was found in cytokine levels of INF-γ. Additionally, only *L. casei* BL23 was able to increase Il-10 levels, compared to the 5-FU inflamed control group (*p* < 0.05).

## Discussion

Mucositis is characterized by inflammation and by cell loss at the level of the epithelial barrier of the digestive tract. This leads to mucosal lesions and/or ulcerations throughout the TGI, i.e., from the mouth to the anus (Soares et al., 2008). Treatments aimed at controlling such side effects of cancer chemotherapy are lacking. We thus proposed to use probiotic bacterial strains, either in suitable culture media (Do Carmo et al., 2019) or in dairy matrices supplemented with whey protein (Cordeiro et al., 2018). We obtained promising results against adverse effects caused by chemotherapeutics, precisely in 5-FU-induced mucositis. Furthermore, Galdindo and collaborators used a FOS prebiotic to attenuate the effects of 5-FU-induced mucositis and obtained promising results (Galdino et al., 2018). Therefore, the present work aimed to develop a symbiotic product from milk, fermented by strains of *Lactobacillus casei* BL23, *Lactiplantibacillus plantarum* B7, and *Lacticaseibacillus rhamnosus* D1, supplemented with WPI, added with FOS, and subsequently lyophilized, to evaluate its therapeutic effects in a murine model of 5-FU-induced mucositis.

Initially, bioactive compounds analyses showed that the Symbiotic (supplemented with FOS) has high levels of the DPPH, ACE, a-amylase, and a-glucosidase inhibitor bioactive compounds. These inhibitor peptides have beneficial effects in other intestinal inflammations such as diabetes, hypertension, obesity, and inflammatory bowel disease (Takiishi et al., 2017; Salinas et al., 2021). It is worth emphasizing the role of the antioxidant activity, which is: protective against histological damage, apoptotic changes, and pro-inflammatory cytokines, mainly caused by chemotherapeutic agents. Given the previous selection of bacterial strains due to their probiotic potential in infection *in vivo* models and mucositis (Acurcio L. B. et al., 2017, Acurcio et al., 2017 LB.; Cordeiro et al., 2018; Valente et al., 2019), and subsequent characterization of the lyophilized fermented product and supplemented with FOS, we decided to investigate its therapeutic potential in a murine model of mucositis induced by 5-FU chemotherapy.

It is known that the application of 5-Fluorouracil in mice leads to a significant weight loss, when compared to non-inflamed animals (Chang et al., 2012). As seen in previous studies, 5-FU-induced mucositis in BALB/c mice triggers a drastic reduction in weight, pasty stools with the presence of blood, as a result of substantial changes in the architecture, and destruction of the intestinal mucosa, resulting in intense inflammatory process (Carvalho RD. et al., 2017; Cordeiro et al., 2018). This also favors a change in intestinal permeability (Galdino et al., 2018; Do Carmo et al., 2019). The probiotic VSL#3 was tested in a murine model of mucositis (Bowen et al., 2007). Hence, this product, which contains several bacterial strains (one strain of *Streptococcus thermophilus*, four *Lactobacillus* spp., and three *Bifidobacterium* spp.), was effective in reducing weight loss in a mucositis model induced by irinotecan. Since mucositis affects the entire gastrointestinal tract, we decided to extend the histological analysis to include the duodenum and jejunum section, together with the ileum section, described in the literature as the site intensely affected by 5-FU action (Touchefeu et al., 2014). In the 5-FU control group, alteration and destruction of the mucosal architecture, as well as extensive inflammation, was observed in the duodenum and jejunum. In the histological analysis of the duodenum, jejunum, and ileum, it was observed that all formulations were able to reduce inflammation in the intestinal mucosa. Interestingly, the Matrix, containing WPI and FOS, was sufficient to reduce inflammation. This result is in agreement with previous studies using WPI and FOS (Cordeiro et al., 2018; Galdino et al., 2018), which indicated significant protection. Other authors suggested that some of the amino acids present in WPI, such as cysteine and glutamate, are used to produce glutathione (Moslehi et al., 2014). This is responsible for providing the main intracellular defense against oxidative stresses, which occurs in severe inflammation such as mucositis, a fact that makes WPI a potential anti-inflammatory compound (Shiby and Mishra, 2013). Futhermore, the results found here using our Symbiotic are similar to those found in other works. For example, Yeung et al., using *Lactobacillus acidophilus* and *Bifidobacterium bifidum* strains, significantly reduced mucosal damage caused by 5-FU-induced mucositis in a murine model (Yeung et al., 2015). In the work of Trindade et al., the administration of the symbiotic Simbioflora^®^, which contains four probiotic strains plus FOS, also reduced damages in the same animal model (Trindade et al., 2018). We suggest that the synergy provided by the interaction of the *Lactobacillus* three strains, with previously tested anti-inflammatory potential, plus FOS, is responsible for improving the response to inflammation. However, more studies will be needed to understand the synergistic mechanisms, the specific action of each of the strains, and the mechanism responsible for mitigation of inflammation caused by chemotherapy.

The impact of mucositis, using chemotherapeutics, expands throughout the intestinal barrier, also affecting the production of goblet cells (Carvalho RDO. et al., 2017). The group that received the lyophilization matrix, interestingly, showed a significant preservation of these cells. Perhaps, the matrix components that were not metabolized by the strains helped in the preservation of goblet cells. A hypothesis for this result, as observed by Cordeiro et al. (2018), would be the availability of amino acids via WPI, mainly threonine, cysteine, ​​and serine for the synthesis of this mucus (Faure et al., 2006) and also the presence of FOS action, increasing the population of Bifidobacteria, which would modulate mucus production.

Mucositis alters the epithelial integrity of the gastrointestinal tract. As a result, the intestinal permeability is affected (Cinausero et al., 2017), allowing translocation of harmful and toxic substances produced by pathobionts bacteria. This may in turn allow their passage from the intestinal lumen to the blood circulation, causing unwanted systemic effects that can lead to death (Fine et al., 2020). Accordingly, 5-FU led here to increased intestinal permeability of animals in the inflamed, when compared to the Naive group. Consumption of *L. casei* BL23 failed to prevent this increase, while preserving the architecture and reducing the inflammation in the small intestine. However, the Matrix and Symbiotic both significantly reduced the intestinal permeability of mucositis mice. In the work of Galdino et al., FOS administration in 5-FU mucositis mice led to similar results (Galdino et al., 2018). In the work of Antunes et al. (2016), the use of the amino acid l-arginine, present in WPI in large quantities, reduced damages to the mucosa and intestinal permeability of animals in a murine model of mucositis. However, synergy with the strains contained here in the Symbiotic promoted an increase in the expression of genes involved in the intestinal epithelial barrier (ZO-1, Ocludin, and Claudin-1). This may explain how this formulation was able to reduce intestinal permeability.

In response to the administration of the chemotherapeutic agent 5-FU, mediators of the inflammatory response are activated, including the transcription factor NF-kb. Its activation leads to the production of pro-inflammatory cytokines, such as TNF-α, IL1-β, and IL-6 and IL-17 (Chang et al., 2012). These inflammatory markers play a central role in mucositis and are released in the inflammatory phase (Lopez-Castejon and Brough, 2011). The symbiotic was able to reduce the level of pro-inflammatory cytokines, which allows us to hypothesize that the therapeutic action of this formulation in the 5-FU-induced mucositis model is mediated by the inhibition of the pro-inflammatory response. This modulatory effect may be favored by metabolites (SCFA, bacteriocins, and neurotransmitters such as GABA) as a result of fermentation by the three strains. This would explain the difference between the Matrix and *L. casei* BL23. However, further experiments will be necessary to fully explain the mechanisms responsible for the healing effect observed here and to identify the different anti-inflammatory effectors produced by these strains.

## Conclusion

In conclusion, we have demonstrated that the lyophilized Symbiotic formulation, containing WPI, FOS, and fermented by *Lactobacillus casei* BL23, *Lactiplantibacillus plantarum* B7 and *Lacticaseibacillus rhamnosus* B1, has anti-inflammatory potential in 5-FU-induced mucositis, reducing animal weight loss, intestinal permeability, modulating genes implicated in the intestinal epithelial barrier, controlling pro-inflammatory cytokine levels, and reducing mucosal damage caused by chemotherapy. This work opens new perspectives for the development of functional symbiotic products for target populations, in the context of mucositis, based on smart selection of matrices and bacterial consortia.

## Data Availability

The raw data supporting the conclusions of this article will be made available by the authors, without undue reservation.
